# Urteilsbildung und Entscheidungsfindung von ASD-Fachkräften in der COVID-19-Krise

**DOI:** 10.1007/s12592-021-00373-6

**Published:** 2021-04-24

**Authors:** Katharina Freres, Megan Benoit, Jana Posmek, Christopher Benkel, Nina Grüßert, Pascal Bastian

**Affiliations:** FB5: Erziehungswissenschaften, Institut für Bildung im Kindes- & Jugendalter, Universität Koblenz-Landau, Bürgerstr. 23, 76829 Landau, Deutschland

**Keywords:** Krise und Bewältigung, COVID-19-Pandemie, Kinder- und Jugendhilfe, Kinderschutz, Ethnographie, Crisis and coping, COVID-19 pandemic, Child and youth welfare, Child protection, Ethnography

## Abstract

Über die Auswirkungen der COVID-19-Pandemie auf die Fallarbeit der Jugendämter liegt bislang noch wenig empirisches Wissen vor. Gleichzeitig sind die konkrete Arbeit der Fachkräfte mit Kindern, Jugendlichen und Familien sowie die Entscheidungspraktiken, etwa bezogen auf den Kinderschutz, auch für die gewöhnliche nicht pandemiebedingte Praxis wenig erforscht. Die in dieser Forschungsnotiz vorgestellte Studie basiert auf einem ethnografischen Forschungsprogramm und untersucht die Fallarbeit in der Pandemie. Aus einer relationalen Perspektive, die den Blick nicht alleine auf die jeweiligen Akteur*innen sondern vielmehr auf deren Verbindungen und Vernetzungen untereinander richtet, sollen Verschiebungen des Netzwerks, in dem die Fälle üblicherweise bearbeitet werden, sichtbar gemacht und Praktiken, die sich als Bewältigungsstrategien der veränderten Praxis fassen lassen, offengelegt werden.

## Abstract (en)

There is still little empirical knowledge about the effects of the coronavirus disease 2019 (COVID-19) pandemic on the case work of youth welfare offices. At the same time, little research has been conducted into the concrete work of the professionals with children, adolescents and families and also into decision-making practices, for example, in relation to child protection, even for ordinary nonpandemic practice. The study is based on an ethnographic research program and examines case work in the pandemic. An assessment from a relational perspective, which focuses not only on the actors, but also on their connections and relations, makes shifts visible in the network in which the cases are usually processed and reveals practices that can be understood as coping strategies for changing practices.

Der weltweit zu beobachtende politische Umgang mit COVID-19 und die damit verbundenen erheblichen Einschränkungen des Alltagslebens, um die Gesundheitssysteme nicht zu überlasten, zeigen sich als ein temporärer Ausnahmezustand (Scherr 2020). Grenzschließungen, Kontaktverbote, Schließung von Sozial- und Bildungseinrichtungen sowie die einschneidenden Maßnahmen bezüglich des sozialen Miteinanders stellen ein bislang global einmaliges und beispielloses Ereignis dar, für das in keinem der betroffenen Systeme eine wirkliche erfahrungs- oder gar evidenzbasierte Umgangsweise verfügbar ist. Davon betroffen sind auch die Kinder- und Jugendhilfe sowie der Kinderschutz – ein Feld, dessen Relevanz gerade in solchen Krisenzeiten mittlerweile medial vermehrt diskutiert und von Expert*innen aus Wissenschaft und Praxis angemahnt wird.

Wie lassen sich in der momentanen, immer wieder als „Krise“ bezeichneten Lage fachliche Arbeit, Kontroll- und Hilfemaßnahmen verwirklichen? Wie lassen sich Arbeitsbündnisse und Vertrauensverhältnisse trotz „Social Distancing“, Quarantäne- und Isolationsbestimmungen aufbauen? Welche Auswirkungen hat dies auf die Arbeit im Kinderschutz und in den Erziehungshilfen, gerade auch hinsichtlich des Potenzials einer Verschärfung familiärer Krisenmomente durch die derzeitige Schließung von Schulen und Krippen, Horten und Kindertageseinrichtungen?

## Forschungsstand oder über das Nicht-Wissen des fachlichen Umgangs mit der „Katastrophe“

Über die Folgen des COVID-19-Lockdowns für Adressat*innen sozialer Dienste und Hilfen wird derzeit vermehrt in den Medien diskutiert, und es finden sich dazu auch einige wenige wissenschaftliche Einschätzungen, die teilweise auf dem empirischen Wissen der Auswirkungen vorangegangener Pandemien, Naturkatastrophen und Ähnlichem basieren.

So verweist Andrew M. Campbell (2020) auf die Auswirkungen der Schließung von Bildungs- und Betreuungseinrichtungen für Opfer von häuslicher Gewalt und Kindesmisshandlung. Lucie Cluver et al. (2020) diskutieren die Herausforderungen, welche sich durch das Wegfallen von Bildungs‑, Freizeit und Betreuungsangeboten gerade für einkommensschwache Familien zeigten. Mit dem Verweis auf die Untersuchungen anderer Krisen, etwa Naturkatastrophen, zeigen Autor*innen wie A. M. Campbell (2020), Jürgen Fegert et al. (2020), L. Cluver et al. (2020) und andere, wie sehr sich dadurch potenzielle Stress- und Risikofaktoren von Gewalt erhöhen.

Gleichzeitig wird aber auch auf die Chance der Krise verwiesen. Caroline Schmitt (2020) spricht gar von einem neuen Auftrag der Sozialen Arbeit, sich im Sinne einer Katastrophenhilfe zu definieren, etwa durch die nachhaltige Gestaltung einer Infrastruktur solidarischer Hilfen, das Finden digitaler Wege, um Hilfen bereitzustellen. Insbesondere jedoch plädiert sie für eine vorausschauende Planung für den Katastrophenfall.

Neben diesem bislang noch überschaubaren Forschungsstand finden sich bereits einige Stellungnahmen. Der Zwischenruf zur COVID-19-Krise der AGJ (2020) etwa mahnt, dass insbesondere der Kinderschutz als „kritische Infrastruktur“ anzuerkennen sei, zu deren Aufrechterhaltung die Jugendämter zurzeit auf Einzelfalllösungen zurückgreifen würden, bezüglich deren Umsetzung noch viele Fragen offen seien. Auch die Erziehungshilfefachverbände in Deutschland (AFET, BvKE, EREV und IGFH) (2020) machen mit ihrem Zwischenruf auf die Sicherstellung des Kinderschutzes in der COVID-19-Krise aufmerksam, so wie auch das Deutsche Jugendinstitut.

Die wenigen empirischen und zum Teil eher programmatischen Beiträge geben wichtige Hinweise über die Herausforderungen, welche die Träger der öffentlichen und freien Kinder- und Jugendhilfe gerade alltäglich meistern müssen. Gleichzeitig offenbaren sie das momentane empirische „Nicht-Wissen“ darüber, wie sich dieser „Katastrophenfall“ auf die alltägliche Handlungspraxis der Fachkräfte auswirkt.

## Untersuchung professioneller Urteilsbildung in der COVID-19 Pandemie. Von neuen „unsichtbaren“ Beteiligten und digitalen Akteur*innen

Die hier vorgestellte Studie schließt an die Ergebnisse des von der Deutschen Forschungsgemeinschaft geförderten Projekts „Fallkonstitutive Urteilsbildung am Beispiel von Kindeswohlgefährdungseinschätzungen bei unangemeldeten Hausbesuchen in der Sozialen Arbeit“ an. Es ist die erste Studie, die konkret vor Ort einen neuralgischen Punkt sozialpädagogischer Fallkonstitution untersucht, nämlich Hausbesuche der Allgemeinen Sozialen Dienste (ASD) (Bastian und Schrödter 2014) und deren Vorgehen bei der Gefährdungseinschätzung im Kinderschutz. In den teilnehmenden Beobachtungen unangemeldeter Hausbesuche von fünf deutschen Jugendämtern konnte eine komplexe Urteilspraxis rekonstruiert werden, bei der im Zentrum steht, dass die Fachkräfte die zu beurteilende Familie unterschiedlichen Bewährungsproben aussetzt (Freres et al. 2019). Über die Beobachtung und Rekonstruktion dieser Testverfahren offenbarte sich eine einfache Heuristik, die erklärt, wie Kinderschutzfachkräfte bei der Verdachtsabklärung zu der Entscheidung gelangen, ob sie einen Fall abschließen können oder eine erzieherische Hilfe vergeben. Die Heuristik zeigt, dass dem Fallverstehen und der Prognose in der realen Urteilspraxis nicht die Bedeutung zufällt, die ihr in der (internationalen) Forschung und in Praxisleitfäden zugemessen wird (Freres 2020). Zugleich offenbart sie, welchen zentralen Stellenwert andere auch *unsichtbare* Akteur*innen in der Urteilspraxis einnehmen. Derart unsichtbare Beteiligte, die bspw. in Erwähnungen oder als Empfänger*innen von Akten und Protokollen „anwesend“ waren, etwa das Familiengericht und die dort urteilenden Richter*innen (Bastian et al. 2017), waren von hoher Relevanz, da Entscheidungen zum Teil bereits zuvor im Hinblick auf diese getroffen wurden.

Damit solche Entscheidungen sich im Urteilsnetzwerk verbreiten können, sind sehr viele nicht-menschliche Akteur*innen nötig, die als Mittler fungieren und eine Transformation der Handlungspraxis der ASD-Fachkräfte z. B. in eine gerichtskompatible Sprache darstellen. Solche Artefakte, die Entscheidungs- und Begründungsdokumentationen zur Verdachtsabklärung enthalten, sind im Besonderen Dokumentationen, Aktennotizen und Diagnosebögen. Mit Verweis auf die Akteur-Netzwerk-Theorie von Bruno Latour (2010) sind solche Objekte relevant, die Handlungen als „Formen“ weiter transportieren und trans*formieren*. Sie handeln mit und weisen sich gegenseitig Rollen zu. Adressat*innen werden zu „Risikofamilien“, Fachkräfte werden zu „Vermittler*innen“ zwischen Kindeswohl und Elternwille oder zu „Übersetzer*innen“ zwischen fachlichen Entscheidungen und gerichtlich verwertbaren Fakten, und Assessment-Tools werden zu „Hilfsmitteln“, um nichts Relevantes zu vergessen.

Was bedeutet diese Rekonstruktion für ein solch etabliertes Handlungs-Netzwerk, wenn sich sehr plötzlich gesellschaftliche Transformationen vollziehen, neue Akteur*innen hinzukommen oder sich Etabliertes verändert? An eben diese Fragen knüpft dieses Forschungsvorhaben an. Die Heuristik mit den Testverfahren erfordert eigentlich eine direkte Interaktion, teilweise eine Konfrontation von Eltern, welche schon durch das unangekündigte Eindringen in den Wohnraum mit der Schwierigkeit konfrontiert werden, ein normales Familienleben zu präsentieren (Freres et al. 2019). Darüber hinaus tritt nun das *Coronavirus* als neuer wichtiger Akteur hinzu.

Die in unseren bisherigen Forschungen herausgestellte Bedeutung verschiedener menschlicher und nicht-menschlicher Akteur*innen zeigt auch, wie unangemessen der Blick auf die professionellen Fachkräfte als exklusive Akteur*innen der Handlungspraxis für die Forschung ist. Wenn wir mit B. Latour (Latour 2010) davon ausgehen, dass sich professionelles Handeln in einem Netzwerk verschiedener (menschlicher wie nicht-menschlicher) Akteur*innen vollzieht (Bastian und Posmek 2020), die auch am Ergebnis einer Handlung beteiligt sind, lohnt es, diesen neuen „Player“ Coronavirus näher als „Mithandelnden“ in den Blick zu nehmen. Mit diesem – so die Vermutung – werden weitere neue Akteur*innen in das Urteilsnetzwerk eingeführt, bzw. die Mittlerfunktion einiger Akteur*innen gestärkt. Das könnten vor allem digitale Akteur*innen sein, wenn etwa Hilfeplanverfahren und Beratungen nicht mehr als Präsenztermin, sondern digital durchgeführt werden müssen.

In der Studie möchten wir diesen Fragen anhand eines ethnographischen Zugangs in verschiedenen, sukzessiv aufeinander aufbauenden Schritten nachgehen. Wir fokussieren uns dabei – wie auch schon in der vorangegangenen Studie – auf die Entscheidungs- und Urteilspraxis der Allgemeinen Sozialen Dienste. Zunächst werden Telefoninterviews mit ASD-Fachkräften von drei bis fünf Jugendämtern durchgeführt, in denen vor allem auf der Grundlage erzählgenerierender Fragen nach den Veränderungen im Arbeitsalltag in der Organisation sowie auf den Einsatz neuer technischer Ressourcen und dessen Auswirkungen auf die Urteilspraxis gefragt wird. In einem zweiten Schritt werden virtuell teilnehmende Beobachtungen der digitalen Beratungs- und Unterstützungspraxis durchgeführt, welche direkte Vergleiche mit den teilnehmenden Beobachtungen der Hausbesuche ermöglichen. In einem dritten Schritt werden zu einem späteren Zeitpunkt – nach der „Krise“ – Anschlussinterviews hinsichtlich der Frage geführt, welche Auswirkungen die veränderten Bedingungen während der Krise auf den Arbeitsalltag und die Nutzung neuer digitaler Tools hatten.

Bei der Auswertung werden der von B. Latour geprägte Netzwerks- und Übersetzungsbegriff sowie die Vorstellung einer Hybridisierung menschlicher und nicht-menschlicher Aktanten eine gewichtige Rolle spielen. Die Auswertung erfolgt auf der Grundlage der von Adele E. Clarke (2011) vorgeschlagenen Reformulierung der Grounded Theory hin zu einer Situationsanalyse. Durch das dort zentrale Verfahren des Mappings erwarten wir eine Vertiefung der Ergebnisse hinsichtlich des verstärkten Einbezugs von Vernetzungen und nicht-menschlichen Akteuren, aber auch von diskursiven Elementen.

Die Ergebnisse des Forschungsvorhabens ermöglichen demnach einen Einblick in den Vollzug von Entscheidungs- und Urteilspraxen im Kinderschutz in Zeiten der COVID-19-Krise, der die Vermutung zulässt, dass bereits rekonstruierte vorhandene Routinen wie die Testverfahren mit einer Krise konfrontiert werden, deren Bewältigung wiederum weitere Erkenntnisse in die Entscheidungs- und Urteilspraxen im Kinderschutz ermöglichen.
